# Obstructive Sleep Apnea, Hypertension, and Their Additive Effects on Atherosclerosis

**DOI:** 10.1155/2015/984193

**Published:** 2015-12-01

**Authors:** Mario Francesco Damiani, Annapaola Zito, Pierluigi Carratù, Vito Antonio Falcone, Elioda Bega, Pietro Scicchitano, Marco Matteo Ciccone, Onofrio Resta

**Affiliations:** ^1^Institute of Respiratory Disease, University of Bari, Bari, Italy; ^2^Section of Cardiovascular Disease, University of Bari, Bari, Italy

## Abstract

*Background and Aims*. It is widely accepted that obstructive sleep apnea (OSA) is independently associated with atherosclerosis. Similar to OSA, hypertension (HTN) is a condition associated with atherosclerosis. However, to date, the impact of the simultaneous presence of OSA and HTN on the risk of atherosclerosis has not been extensively studied. The aim of this study was to evaluate the consequences of the coexistence of OSA and HTN on carotid intima-media thickness (IMT) and on inflammatory markers of atherosclerosis (such as interleukin- [IL-] 6 and pentraxin- [PTX-] 3).* Methods*. The study design allowed us to define 4 groups: (1) controls (*n* = 30); (2) OSA patients without HTN (*n* = 30); (3) HTN patients without OSA (*n* = 30); (4) patients with OSA and HTN (*n* = 30). In the morning after portable monitoring (between 7 am and 8 am), blood samples were collected, and carotid IMT was measured.* Results*. Carotid IMT, IL-6, and PTX-3 in OSA normotensive patients and in non-OSA HTN subjects were significantly higher compared to control subjects; in addition, in OSA hypertensive patients they were significantly increased compared to OSA normotensive, non-OSA HTN, or control subjects.* Conclusions*. OSA and HTN have an additive role in the progression of carotid atherosclerosis and in blood levels of inflammatory markers for atherosclerosis, such as interleukin-6 and pentraxin-3.

## 1. Introduction

Obstructive sleep apnea (OSA) is a disorder characterized by recurrent episodes of upper airway obstruction during sleep [1], resulting in chronic intermittent hypoxia and sleep fragmentation [2–4]. It is widely accepted that OSA is independently associated with atherosclerosis [5, 6]. Indeed, among OSA patients, an increase in carotid intima-media thickness (IMT), a validated marker of atherosclerosis, has been largely demonstrated [7–12]. Moreover, our group recently observed the reversibility of the endothelial dysfunction, as measured by flow-mediated dilation (FMD), after treatment with continuous positive airway pressure (CPAP) [13]. On the other hand, there is evidence that OSA is associated with increased blood levels of inflammatory markers, such as interleukin- (IL-) 6 and pentraxin- (PTX-) 3 [14–16], which are also considered as important indicators for atherosclerosis, on the basis of several studies [17–20]. However, it is important to underline that OSA is commonly associated with several comorbidities. In this regard, there is a close relationship between OSA and hypertension (HTN) [21]. In patients with OSA, the prevalence of HTN is about 50–60%, and the presence of HTN seems to be a consequence of both environmental and genetic factors [22]. Similar to OSA, HTN is a condition associated with atherosclerosis [23]. However, to date, the impact of the simultaneous presence of OSA and HTN on the risk of atherosclerosis has not been extensively studied.

The aim of this study was to evaluate the consequences of the coexistence of OSA and HTN on a marker of carotid atherosclerosis (such as IMT) and on inflammatory markers of atherosclerosis (such as IL-6 and PTX-3).

## 2. Materials and Methods

### 2.1. Patients and Study Design

During a period of eighteen months, we recruited patients who were referred to our Sleep Laboratory with suspected OSA. Moreover, we selected healthy subjects and patients with a diagnosis of HTN at low risk for OSA (based on a comprehensive anamnestic symptom evaluation/physical examination [24]) to undergo in-laboratory portable monitoring (PM). After PM, only subjects with no OSA (apnea-hypopnea index [AHI] < 5) or with moderate-severe OSA (AHI ≥ 15) were included in the study. The study design allowed us to define 4 groups: (1) controls (*n* = 30); (2) OSA patients without HTN (*n* = 30); (3) HTN patients without OSA (*n* = 30); (4) patients with OSA and HTN (*n* = 30). Groups were matched for age (±5 years) and body mass index (BMI, ±2.5 kg/m^2^). Exclusion criteria were as follows: chronic obstructive pulmonary disease (COPD), history of smoking, congestive heart failure (CHF), previous myocardial infarction, unstable angina, prior coronary intervention, arrhythmias, use of cardioactive drugs, chronic renal disease, diabetes mellitus, morbid obesity (BMI > 40 kg/m^2^), any chronic inflammatory disease, and systemic infections at the time of the study or within two weeks before the study. HTN patients were outpatients from the Section of Cardiovascular Disease, and HTN was previously diagnosed according to current guidelines [21]. The cutoff point for HTN was 140/90 mmHg [21]. Blood pressure (BP) was measured using a sphygmomanometer with an appropriately sized cuff. Subjects had their BP measured while being seated after 5 min of rest. Three consecutive measurements were carried out and mean of last two BP values was recorded. For ethical reasons, all the HTN patients were on medications for blood pressure control. In the morning after PM (between 7 am and 8 am), blood samples were collected, and carotid IMT was measured. In order to avoid acute effects of antihypertensive treatment, patients did not receive medications on the day IMT was measured, as previously described [25–27].

The study was approved by the Institutional Review Board of Bari University General Hospital and carried out in accordance with the principles of the Helsinki Declaration. All patients gave prior written informed consent to take part in the study.

### 2.2. Portable Monitoring

All subjects underwent overnight in-laboratory portable monitoring with the Somtè Compumedics Inc., Abbotsford, VIC, Australia. Two sleep medicine physicians interpreted PM recordings [14, 28]. The diagnosis of OSA was based on apnea-hypopnea index (AHI) ≥ 5 events/h, which was further subdivided into mild (5 ≤ AHI < 15 events/h) and moderate-severe (≥15 events/h). As in our previous works, in order to obtain the useful recording time, patients were asked to fill out a sleep diary [14, 29]. The Epworth Sleepiness Scale was performed to assess daytime sleepiness.

### 2.3. Carotid IMT

All patients underwent two-dimensional echo-color Doppler of the carotid arteries, adopting a high definition vascular echograph Philips Sonos 5500 Bothell, Washington, USA, and a 10–3 MHz linear electronic probe. All subjects were examined by the same investigator. IMT is defined as a double-line pattern visualised ecographically on both walls of the common carotid artery in a longitudinal view. Two parallel lines (leading edges of two anatomical boundaries) form it: lumen-intima and media-adventitia interfaces [30, 31]. As in our previous works, the IMT of the far wall of the right common carotid artery on the lengthwise axis was reported [7, 32]. Mean IMT value (m-IMT) was obtained from measurements made in three key points: proximal (~2 cm before the flow-divider), distal (~1/2 cm before the flow-divider), and middle zone [7].

### 2.4. Measurement of Inflammatory Markers

Samples of peripheral venous blood were collected between 7 a.m. and 8 a.m. Samples were stored at −80°C until the time of assay. Quantitative sandwich enzyme immunoassay kits (R&D Systems) were used to measure IL-6 and PTX-3 concentrations in plasma.

### 2.5. Statistical Analysis

Data are presented as mean ± SD unless otherwise indicated. Differences between four groups were analyzed by analysis of variance with Bonferroni correction. Multiple regression analysis was performed to identify variables that were independently associated with carotid IMT, IL-6, and PTX-3. A value of *p* < 0.05 was considered statistically significant. The analyses were made using STATISTICA 6.1 software (StatSoft Inc., Tulsa, Oklahoma).

## 3. Results

Characteristics of study population including age, sex, body mass index, neck circumference, systolic/diastolic blood pressure, heart rate, apnea-hypopnea index, total sleep time with oxyhemoglobin saturation below 90% (TST90), and SaO_2_ nadir are shown in Table 1. Systolic and diastolic blood pressure were significantly higher in HTN and OSA + HTN groups than in controls and OSA groups. AHI and TST90 were significantly higher in OSA and OSA + HTN groups than in controls and HTN groups; SaO_2_ nadir was significantly lower in OSA and OSA + HTN than in controls and HTN groups. In the hypertension and the OSA-plus-hypertension groups, the percentage of patients who were receiving diuretics, *β*-blockers, calcium-channel blockers, angiotensin-converting enzyme inhibitors, and angiotensin II receptor antagonists was similar (data not shown).

**Figure 1:**
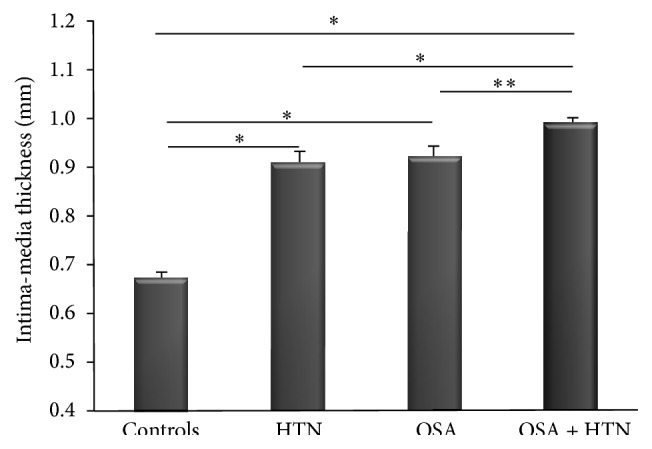
Carotid IMT in controls, HTN, OSA, and OSA + HTN subjects. ^*∗*^ *p* < 0.01; ^*∗∗*^ *p* < 0.05.

Carotid IMT values are shown in Figure 1. Carotid IMT in OSA normotensive patients (0.92 ± 0.09 mm) and in non-OSA HTN subjects (0.90 ± 0.08 mm) was significantly higher compared to control subjects (0.67 ± 0.1 mm; *p* < 0.01). In addition, carotid IMT in OSA hypertensive patients (0.99 ± 0.09 mm) was significantly increased compared to OSA normotensive subjects (*p* < 0.05), non-OSA HTN subjects (*p* < 0.01), or control subjects (*p* < 0.01).

**Figure 2:**
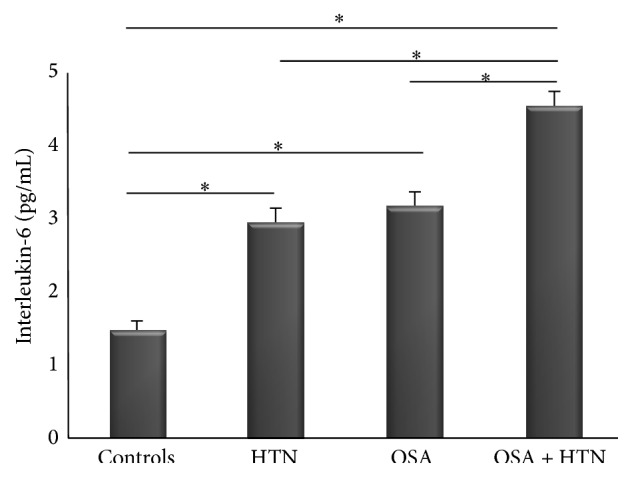
IL-6 in controls, HTN, OSA, and OSA + HTN subjects. ^*∗*^ *p* < 0.01.

**Figure 3:**
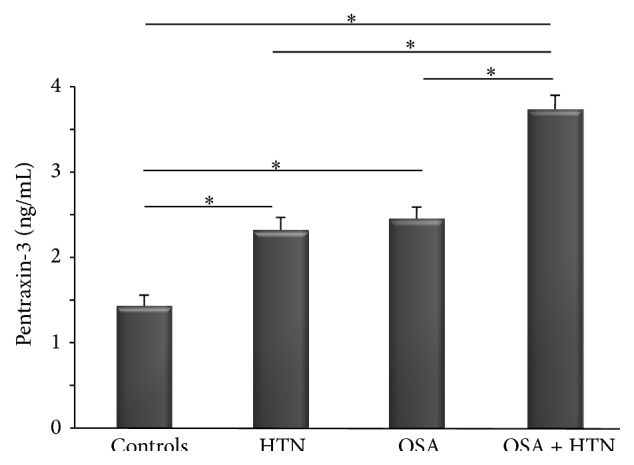
PTX-3 in controls, HTN, OSA, and OSA + HTN subjects. ^*∗*^ *p* < 0.01.

Plasma levels of interleukin-6 and pentraxin-3 are shown in Figures 2 and 3, respectively. Levels of IL-6 and PTX-3 in OSA normotensive patients (3.14 ± 1.27 pg/mL, 2.46 ± 0.43 ng/mL, resp.) and in non-OSA HTN patients (2.92 ± 1.21 pg/mL, 2.31 ± 0.41 ng/mL, resp.) were significantly higher compared to control subjects (1.46 ± 0.69 pg/mL, *p* < 0.01; 1.44 ± 0.43 ng/mL, *p* < 0.01, resp.). Moreover, levels of IL-6 and PTX-3 in OSA + HTN patients (4.51 ± 1.34 pg/mL, 3.75 ± 0.48 ng/mL, resp.) were significantly elevated compared to OSA normotensive subjects (*p* < 0.01 for both markers), non-OSA HTN subjects (*p* < 0.01 for both markers), or control subjects (*p* < 0.01 for both markers).

In the multivariate analysis performed on the whole study sample, carotid IMT, PTX-3, and IL-6 were used as dependent variables, and their relationship with the following independent variables was evaluated: age, BMI, neck circumference, systolic/diastolic blood pressure, heart rate, AHI, TST90, and SaO_2_ nadir. Carotid IMT was positively related to AHI and systolic blood pressure (*p* < 0.05). The only variable independently associated with IL-6 was AHI (*p* < 0.01). The two variables independently associated with PTX-3 were AHI and systolic blood pressure (*p* < 0.01).

## 4. Discussion

The present study demonstrated that carotid IMT, interleukin-6, and pentraxin-3 have a similar increase among OSA normotensive and non-OSA HTN patients, compared to normotensive non-OSA subjects. In addition, a further increase of the three above-mentioned atherosclerotic markers in OSA HTN patients was found. Further indicating the role of both OSA and HTN it was observed that AHI and systolic blood pressure were independently associated with carotid IMT and inflammatory markers. These results lead to considering a possible additive role of OSA and HTN on the progression of endothelial dysfunction.

To date, in the literature two important works are reported, in which the role of OSA + HTN on the progression of atherosclerosis has been evaluated. Drager et al. [25] studied 60 middle-aged subjects classified into four groups according to the presence of OSA or not, with and without HTN; they found an increase in arterial stiffness, evaluated by pulse-wave velocity, among OSA hypertensive patients. More recently, the same group [33] studied the impact of the coexistence of OSA and HTN on early markers of carotid atherosclerosis; they demonstrated additive effects of OSA and HTN on carotid IMT, diameter, and distensibility. According to these results, we found increased carotid arterial stiffness among OSA hypertensive patients, compared to patients without either condition.

Pathophysiologically, it is widely accepted that the role of both OSA and HTN on the progression of endothelial damage is mediated by inflammation [34–36]. Indeed, in patients with HTN, increased blood pressure triggers inflammatory mechanisms, in which vasoactive peptides angiotensin II and endothelin-1 have an important role [36]. On the other hand, in OSA patients, the hypoxia/reoxygenation cycle and sleep fragmentation promote generation of reactive oxygen species and inflammation [34]. To date, several studies evaluated blood levels of two important inflammatory markers for atherosclerosis, such as IL-6 and PTX-3, among patients with HTN or OSA alone [14, 16, 37, 38]. These works demonstrated a relationship between the presence of OSA/HTN and increased values of these two inflammatory markers [14, 16, 37, 38]. However, blood levels of IL-6 and PTX-3 have never been evaluated among subjects with both conditions. We found increased values of these inflammatory markers in OSA HTN patients, compared to OSA normotensive or non-OSA HTN subjects. Given the largely known role of IL-6 and PTX-3 as indicators for atherosclerosis [17–20], our results (also including the increased carotid IMT in OSA HTN subjects) suggest an additive effect of OSA and hypertension on the progression of endothelial impairment. Indeed, it is conceivable that mechanisms responsible for both inflammation and endothelial dysfunction in patients with OSA or HTN alone may overlap in presence of both conditions, resulting in a more pronounced progression of atherosclerosis. With regard to interleukin-6, there is evidence that it is a proinflammatory cytokine which plays an important role in the pathogenesis of atherosclerosis [39]. Differently, there are still lights and shadows concerning the relationship between pentraxin-3 and endothelial impairment. On one hand, there is evidence that PTX-3 is a specific marker for endothelial inflammation, due to its production, in response to proinflammatory signals, predominantly by macrophages and vascular endothelial cells [16]. On the other hand, the role of pentraxin-3 in pathogenesis of vascular pathology is still debated: some studies suggest a pathogenic role of PTX-3 in atherosclerosis [40], while other works lead to hypothesizing an atheroprotective effect [41]. However, whether the increased levels of PTX-3 observed in patients with OSA + HTN are an epiphenomenon of the atherosclerotic process or whether the protein has an active role in development of endothelial dysfunction is beyond the scope of the present study. Further studies are needed to clarify the molecular mechanisms that link PTX-3 to atherosclerosis.

Some important limitations need to be considered. First, although OSA patients were not receiving treatment, all the HTN subjects were in treatment (though their mean systolic/diastolic blood pressure was significantly higher than in normotensive subjects). On the other hand, it is well known that HTN patients have increased endothelial impairment compared to normotensive patients, despite optimal treatment [42]. This consideration, together with the results of the present study, leads to considering our study design as valid and reliable. Second, all the subjects with mild OSA were excluded from the study, and therefore our study population is not representative of all the OSA population. However, some previous studies showed the absence of significant differences in terms of endothelial dysfunction/inflammation between mild OSA and healthy subjects [16]. Thus, we considered the presence of mild OSA as a confounding factor, and therefore we excluded a priori these subjects. Another important limitation in the present study is the lack of ambulatory blood pressure data.

In conclusion, OSA and HTN have an important pathogenic role in vascular pathology. Moreover, these two conditions have an additive role in the progression of carotid atherosclerosis and in blood levels of inflammatory markers for atherosclerosis, such as interleukin-6 and pentraxin-3.

## Figures and Tables

**Table 1:** Demographic and polygraphic characteristics of study population.

	Controls	OSA	HTN	OSA + HTN
Subjects, *n*	30	30	30	30
Age, years	52.70 ± 10.66	53.46 ± 9.96	52.50 ± 9.89	52.46 ± 10.90
Sex, male/female	24/6	26/4	25/5	27/3
BMI, kg/m^2^	28.37 ± 2.80	29.10 ± 2.62	28.64 ± 2.67	29.24 ± 2.47
Neck circumf., cm	39.76 ± 2.99	40.46 ± 2.86	39.86 ± 2.58	40.63 ± 2.60
Blood pressure, mmHg				
Systolic	122.66 ± 11.72	125.16 ± 10.54	141.16 ± 15.18^#^	142.66 ± 15.01^#^
Diastolic	73.66 ± 9.09	74.33 ± 9.35	83.66 ± 10.98^#^	84.16 ± 11.60^#^
Heart rate, bpm	71.20 ± 9.98	74.93 ± 11.06	73.86 ± 10.90	74.40 ± 10.64
AHI, events/h	2.12 ± 1.21	43.14 ± 14.04^*∗*^	1.79 ± 1.14	45.27 ± 15.13^*∗*^
TST90, %	0.05 ± 0.09	28.66 ± 12.37^*∗*^	0.04 ± 0.08	29.34 ± 11.98^*∗*^
SaO_2_ nadir, %	88.73 ± 3.60	73.06 ± 8.80^*∗*^	88.20 ± 3.96	72.76 ± 7.54^*∗*^

Data are presented as mean values ± SD or as number. OSA, obstructive sleep apnea, HTN, hypertension, BMI, body mass index, AHI, apnea-hypopnea index, TST90, total sleep time with oxyhemoglobin saturation below 90%, and SaO_2_, arterial oxygen saturation.

^#^Data are different from controls and OSA groups (*p* < 0.01).

^*∗*^Data are different from controls and HTN groups (*p* < 0.01).
